# MCL-1ES Induces MCL-1L-Dependent BAX- and BAK-Independent Mitochondrial Apoptosis

**DOI:** 10.1371/journal.pone.0079626

**Published:** 2013-11-18

**Authors:** Jae-Hong Kim, Jeehyeon Bae

**Affiliations:** College of Pharmacy, Chung-Ang University, Seoul, Korea; Osaka University Graduate School of Medicine, Japan

## Abstract

MCL-1 (myeloid cell leukemia-1), a member of the BCL-2 family, has three splicing variants, antiapoptotic MCL-1L, proapoptotic MCL-1S, and MCL-1ES. We previously reported cloning MCL-1ES and characterizing it as an apoptotic molecule. Here, we investigated the molecular mechanism by which MCL-1ES promotes cell death. MCL-1ES was distinct from other proapoptotic BCL-2 members that induce apoptosis by promoting BAX or BAK oligomerization, leading to mitochondrial outer membrane permeabilization (MOMP), in that MCL-1ES promoted mitochondrial apoptosis independently of both BAX and BAK. Instead, MCL-1L was crucial for the apoptotic activity of MCL-1ES by facilitating its proper localization to the mitochondria. MCL-1ES did not interact with any BCL-2 family proteins except for MCL-1L, and antiapoptotic BCL-2 members failed to inhibit apoptosis induced by MCL-1ES. The BCL-2 homology 3 (BH3) domain of MCL-1ES was critical for both MCL-1ES association with MCL-1L and apoptotic activity. MCL-1ES formed mitochondrial oligomers, and this process was followed by MOMP and cytochrome *c* release in a MCL-1L-dependent manner. These findings indicate that MCL-1ES, as a distinct proapoptotic BCL-2 family protein, may be useful for intervening in diseases that involve uncontrolled MCL-1L.

## Introduction

Apoptosis, evolutionally conserved programmed cell death, is essential to maintain cellular homeostasis, and its aberrant regulation leads to a variety of disorders. BCL-2 family members are central regulators of apoptosis in diverse species and comprise both antiapoptotic and proapoptotic subfamilies, which are classified based on their structures and functions [1]. Antiapoptotic BCL-2 subfamily members include BCL-2, MCL-1, BCL-xL, BCL-w, and BFL1 and promote cell survival by sequestering proapoptotic BCL-2 members, such as BAX, BAK, BIM, BID, BAD, NOXA, PUMA, and HRK, through partner-specific interactions [2]. Proapoptotic BCL-2 subfamily proteins are further classified as either BCL-2 homology 3 (BH3) domain-only members or multi-domain BAX and BAK proteins. Upon receiving death signals, BAX and BAK undergo extensive conformational changes to form oligomers and induce mitochondrial outer membrane permeabilization (MOMP), which leads to the release of apoptotic molecules, including cytochrome *c*, and subsequent caspase activation [3]. Proapoptotic BH3-only proteins activate BAX and/or BAK at the outer mitochondrial membrane, and their apoptotic effects are inhibited by the antiapoptotic members of the BCL-2 family [4]. Thus, a delicate stoichiometric balance between anti- and proapoptotic BCL-2 family members determines a cell’s fate, in which the heterodimerization between BCL-2 family proteins via the BH3 domains, amphipathic α-helixes, is a regulatory cue.

MCL-1, an antiapoptotic BCL-2 family member, is a crucial survival-promoting molecule for a variety of cell types [5]. Three alternative splicing variants of the human *MCL-1* gene, MCL-1L, MCL-1S, and MCL-1ES, have been identified [6]–[8]. We have reported that exons I to III of *MCL-1* encode the pro-survival MCL-1L protein, while two splicing events produce the cell death-inducing proteins MCL-1S and MCL-1ES [7], [8]. MCL-1ES induces mitochondrial cell death and dimerizes with MCL-1L [8]. However, the underlying molecular mechanism by which MCL-1ES induces apoptotic cell death remains unknown. In this study, we identified a unique cell death mechanism for the MCL-1ES protein: MCL-1ES induces BAX- and BAK-independent apoptosis, MCL-1ES forms mitochondrial oligomers, and MCL-1L is crucial for the apoptotic activity of MCL-1ES.

## Materials and Methods

### Chemicals

The chemicals used in the experiments were purchased from Sigma (St. Louis, MO, USA) unless otherwise indicated.

### Plasmid Construction

Cloning of pcDNA3 Flag-MCL-1ES and MCL-1L was reported previously [7], [8]. pCMV Myc-tagged (Clontech, Mountain View, CA, USA) MCL-1ES was cloned after PCR amplification using the following primers: MCL-1ES-F (5′-CTAGAATTCAAATGTTTGGCCTCAA) and MCL-1ES-R (5′-CTAGCG GCCGCCTATCTTTTTAGAT). The following mutant forms of MCL-1ES were cloned into pCMV-Myc after PCR amplification using the following primer sets: MCL-1ES (amino acids 1–173) by MCL-1ES-F and MCL-1ES 173-R (5′-CTAGCGGCCGCCTAACCTTCTAGGTC), MCL-1ES (amino acids 1–76) by MCL-1ES-F and MCL-1ES 76-R (5′-CTAGCGGCCGCCTAGAAGGCCGTCTC), MCL-1ES (amino acids 78–197) by MCL-1ES 78-F (5′- CTAGAATTCAAATGCTTCGGAGACTG) and MCL-1ES-R, and MCL-1ES (amino acids 78–173) by MCL-1ES 78-F and MCL-1ES 173-R. The pCMV-Myc MCL-1ES BH3M construct was produced by a recombinant PCR technique using the following primers: MCL-1ES-F, MCL-1ES BH3 M-R (5′-GAGCACCAACGCGACGTGACGACGACGACGACGACGACGCCAGAGGTCGCGGAAGGACGA), MCL-1ES BH3 M-F (5′-GGTCTCCAGCGCCTTCCTGCT), and MCL-1ES-R. The pET-28a(+) (Novagen, San Diego, CA, USA) MCL-1L and MCL-1ES constructs were produced after PCR amplification using the following primers: pET-28a(+) MCL-1-F (5′-CTACTACATATTCAAATGTTTGGCCTCAA) and pET-28a(+) MCL-1-R (5′-TAGAAGCTTCTATCTTTTTAGAT).

### Cell Culture and Transfection

293T cells (ATCC, Manassas, VA, USA) and mouse embryo fibroblasts (MEFs) were cultured in Dulbecco’s modified Eagle’s medium (DMEM) (Gibco, Paisley, Renfrewshire, UK). Wild-type, *bax* ^−/−^, *bak* ^−/−^, and *bax* ^−/−^ *bak* ^−/−^ MEF cells [9] were generous gifts from Dr. CB Thompson (University of Pennsylvania, PA, USA). *Bim* ^−/−^, *Noxa* ^−/−^, and *Puma* ^−/−^ MEF cells [10], [11] were kindly provided by Dr. Strasser (The Walter and Eliza Hall Institute of Medical Research, Melbourne, Australia). Cells were transfected as previously described [12].

### Cell Viability Assay

Twenty-four hours after transfection, cell viability was measured using a Cell Titer-Glo assay kit (Promega, Madison, WI, USA). Briefly, 96-well plates containing cells (1.0×10^5^) were removed from the incubator and allowed to equilibrate to room temperature for 5 minutes. Cell Titer-Glo reagent was added to each well, and samples were mixed with a plate shaker for 5 min. The plate was then incubated at room temperature for 30 min. The luminescence of each sample was measured in a microplate reader (FlexStation 3; Molecular Devices, Sunnyvale, CA, USA) with a 1 second/well read time.

### Flow Cytometry Analysis of Annexin V-positive Cells

Cells were cultured in 6 cm culture dishes for 24 h, transfected with 3 µg of various pcDNA3 plasmid constructs, harvested with 0.5 mM EDTA, and washed with PBS (137 mM NaCl, 2.7 mM KCl, 10 mM Na_2_HPO_4_, and 2 mM KH_2_PO_4_). Binding buffer (0.1 M HEPES at pH 7.4, 1.4 M NaCl, and 25 mM CaCl_2_) was added to the cells, and an aliquot was transferred to a new tube containing Annexin V-FITC (BD Pharmingen, San Diego, CA, USA). After incubating for 1 h on ice in the dark, the cells were washed once with PBS, and the binding buffer was added. Propidium iodide (PI; BD Pharmingen) was added to the cells before running FACSCalibur (BD Biosciences, Franklin Lakes, NJ, USA).

### Measurement of Membrane Permeabilization (MMP)

MMP was measured using JC-1 and flow cytometric analysis with a FACSCaliber as reported previously [8].

### Determination of Caspase Activation

Cells (1.0×10^6^) were plated onto 60 mm dishes and transfected with a total of 3 µg of the appropriate plasmid DNA. After 24 h, the cells were lysed, and the lysates were subjected to electrophoresis and immunoblotted with anti-caspase 3 (Cell Signaling, Danvers, MA, USA: #9662), anti-caspase 9 (Cell Signaling: #9508), and anti-caspase 8 antibodies (Cell Signaling: #4790).

### Caspase 3 Activity Assay

The activity of caspase 3 was measured using a fluorometric kit (R&D System, Minneapolis, MN, USA) according to the manufacturer’s instructions. Briefly, cells were harvested using caspase lysis buffer (50 mM HEPES, pH 7.4, 0.1% CHAPS, 5 mM dithiothreitol, 0.1 mM EDTA, and 0.1% Triton X-100), and the cell lysate samples (20 µg) were used. The fluorescence emission of 7-amino-4-trifluoromethylcoumarin (AFC), released upon proteolytic cleavage of the fluorogenic substrate DEVD (Asp-Glu-Val-Asp)-AFC by active caspase 3, was measured using FlexStation 3 (excitation wavelength, 400 nm; emission wavelength, 505 nm).

### Mitochondrial Fractionation

Mitochondria were isolated using a mitochondria isolation kit (Thermo Scientific, Rockford, IL, USA) according to the manufacturer’s instructions. Mitochondria were resuspended in isotonic buffer (0.2 M mannitol, 50 mM sucrose, 1 mM EDTA, and 20 mM HEPES-KOH, pH 7.4), treated with 50 µg/ml trypsin for 30 min on ice, and analyzed by western blotting.

### Cytochrome c Release

Using a digitonin-based method that we described previously [8], the cytosolic and heavy membrane fractions were separated and analyzed by western blotting with the appropriate antibodies.

### Expression and Purification of the Recombinant MCL-1L and MCl-1ES Proteins

The recombinant MCL-1L and ES proteins were produced according to a previous report [13].

### Cell-free Cytochrome c Release Assay

Mitochondria (0.05 mg) isolated from 293T cells were added to 0.1 ml of release buffer (210 mM mannitol, 70 mM sucrose, 10 mM HEPES : NaOH, pH 7.4, 0.5 mM EGTA, 5 mM succinate, 4 mM MgCl_2_, and 5 mM Na_2_HPO_4_). The purified recombinant monomeric, full-length MCL-1ES protein (0.01 µg) was added in the absence or presence of purified recombinant MCL-1L and was incubated for 30 min at RT. The presence of cytochrome *c* was analyzed in the pellet and supernatant by western blotting using cytochrome *c* antibodies.

### Confocal Microscopic Immunofluorescence Analysis

Twenty-four hours after transfection, 293T cells were fixed with 4% paraformaldehyde, permeabilized with 0.2% Triton-X-100, and incubated with blocking buffer (PBS containing 2% FBS and 0.01% NaN_3_). The cells were then incubated with antibodies in PBS containing 0.1% Tween 20. To visualize the mitochondria, the cells were incubated with MitoTracker (Invitrogen, Carlsbad, CA, USA) for 15 minutes before fixation. To detect Flag-MCL-1ES, the cells were incubated with anti-Flag polyclonal antibodies (Sigma) and Alexa Fluor 488 goat anti-rabbit IgG (Invitrogen). Fluorescence was detected using a Zeiss LSM 510 META confocal microscope (Carl Zeiss, Gottingen, Germany).

### Protein Oligomerization

The digitonin-permeabilized cells were analyzed as described elsewhere [12].

### Immunoprecipitation

Immunoprecipitation and western blot analyses were conducted according to our previous report [8]. Proteins were detected by anti-MCL-1L (Santa Cruz Biotechnology, Santa Cruz, CA, USA), anti-Flag (Sigma), and anti-Apaf1 antibodies (Santa Cruz Biotechnology).

### Yeast Two-hybrid Assay

The interactions between human MCL-1ES and other BCL-2 family members were assessed using a GAL4-based yeast two-hybrid system (Clontech) as described previously [7].

### Statistical Analysis

Multiple comparison analyses of values were performed with the Student-Newman-Keuls test (SAS, Cary, NC, USA).

## Results

### The Crucial Role of the MCL-1ES N-terminus for MCL-1ES Apoptotic Activity

**Figure 1:**
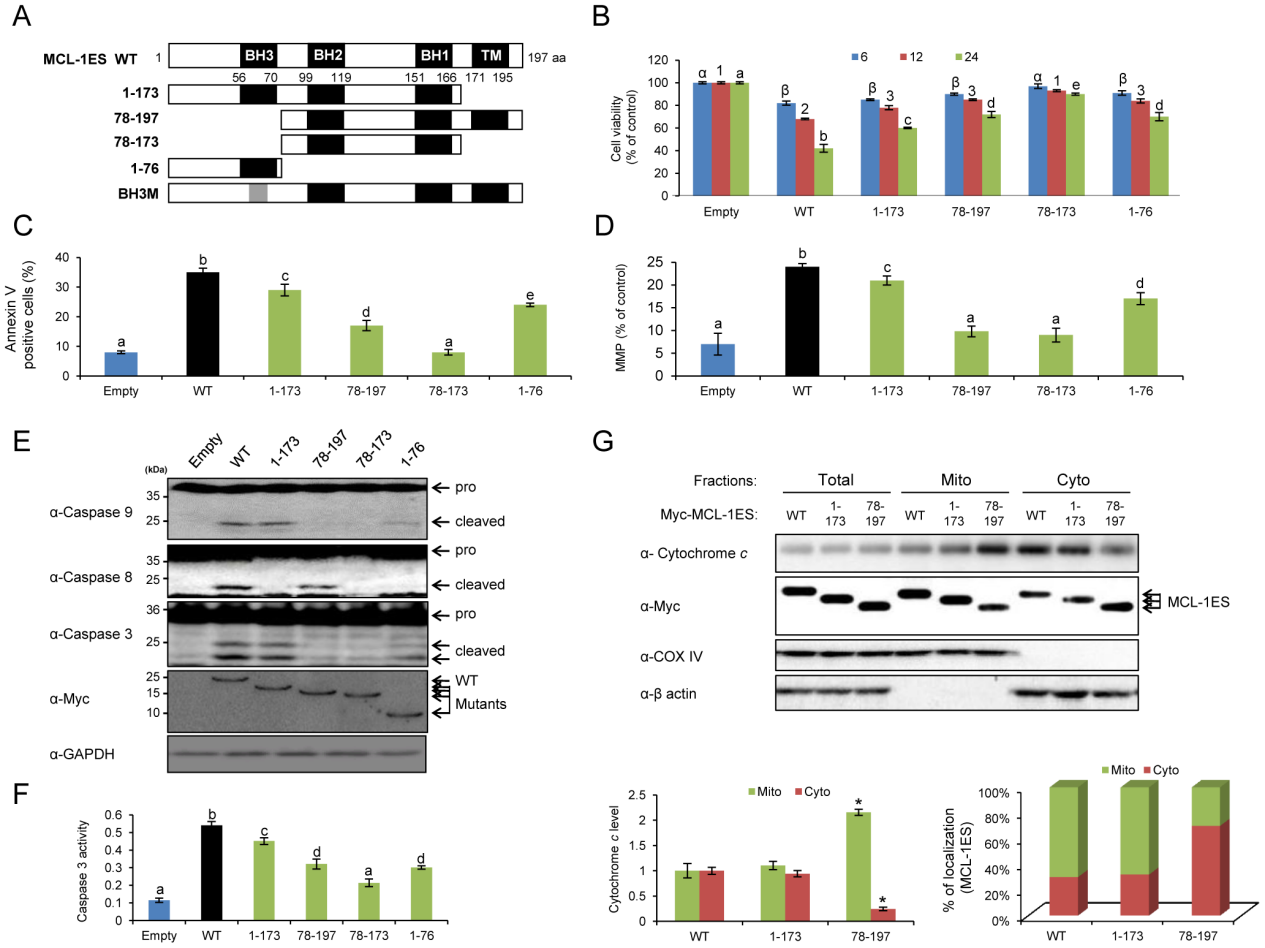
Mapping the MCL-1ES domains crucial for mitochondrial apoptosis. (A) The MCL-1ES mutants generated are shown. (B) A time course (6, 12, and 24 h) of cell viability of the MCL-1ES mutants in 293T cells is shown after transfecting equal amounts of the respective constructs. (C) Flow cytometry analysis of Annexin V-positive apoptotic cells was performed and (D) MMP of 293T cells was determined 24 h after transfection. Three independent experiments were performed, and the data are expressed as the mean ± SEM. Different letters denote statistically significant different values (*P*>0.05). (E) The activation of caspases 9, 8, and 3 was assessed by immunoblot analysis using transfected 293T cells. (F) Caspase 3 activity was measured using the DEVD peptide conjugated to *p*-nitroaniline as described in the Material & Methods. The values are expressed as the mean ± SEM of three replicates. (G) The cytosolic release of cytochrome *c* was assessed in transfected 293T cells. The cells were separated into mitochondrial and cytosolic fractions. Adequate fractionation was demonstrated by immunoblot analysis using anti-cox IV and anti-β-actin antibodies. The relative cytochrome *c* level (left graph) was quantified, and MCL-1ES localization (right graph) in the mitochondrial and cytoplasmic fractions was presented (lower panel). The values are expressed as the mean ± SEM of three independent experiments.

To identify the domain of MCL-1ES required for its cell death activity, we generated truncated mutants of MCL-1ES (Figure 1A). Compared to wild-type (WT) MCL-1ES (amino acids 1–197), deleting the *C-*terminus region (leaving amino acids 1–173), which contains the transmembrane domain (TM), moderately attenuated cell death, Annexin V-positive apoptotic cells, MMP, and caspase activation (Figure 1B–F). Incubation with increasing concentrations of z-VAD-fmk, a pan-caspase inhibitor, attenuated the cell death induced by MCL-1ES (Figure S1). Truncating the *N*-terminus, which encompasses the BH3 domain (leaving amino acids 78–197), greatly diminished apoptosis and shifted MCL-1ES localization to the cytoplasm compared to WT (Figure 1B–G). Deleting both the BH3 and TM domains of MCL-1ES (leaving amino acids 78–173) resulted in the loss of the apoptotic effect, while a short peptide retaining the BH3 domain (containing amino acids 1–76) possessed cell death activity (Figure 1B–F). Interestingly, MCL-1ES mutants that possess the BH3 domain but lack the TM domain (containing amino acids 1–173 or 1–76) activated caspases 9 and 3 but were unable to activate caspase 8 (Figure 1E), implying that two distinctive pathways are likely to be involved: BH3 domain-dependent mitochondrial oligomer formation-mediated caspase 9 activation and TM-dependent caspase 8 activation. Furthermore, MCL-1ES mutations changed the cytosolic release of mitochondrial cytochrome *c*. Consistent with the apoptotic events shown in Figure 1B–F, the mutant with amino acids 1–173 induced a similar degree of cytochrome *c* release into the cytoplasm compared to WT MCL-1ES, whereas cytochrome *c* release by the mutant containing amino acids 78–197 showed reduced cytochrome *c* release (Figure 1G: left graph). Ectopically expressed MCL-1ES and the variant of the protein that contained amino acids 1–173 were mainly present in the mitochondrial fraction, but the variant containing amino acids 78–197 and lacking the BH3 domain was predominantly detected in the cytoplasm (Figure 1G: left graph). These results indicate that the *N*-terminus of MCL-1ES, including the BH3 domain, is crucial for its mitochondrial localization and mitochondrial apoptotic activities.

### BH3 Domain-dependent Apoptosis by MCL-1ES

**Figure 2:**
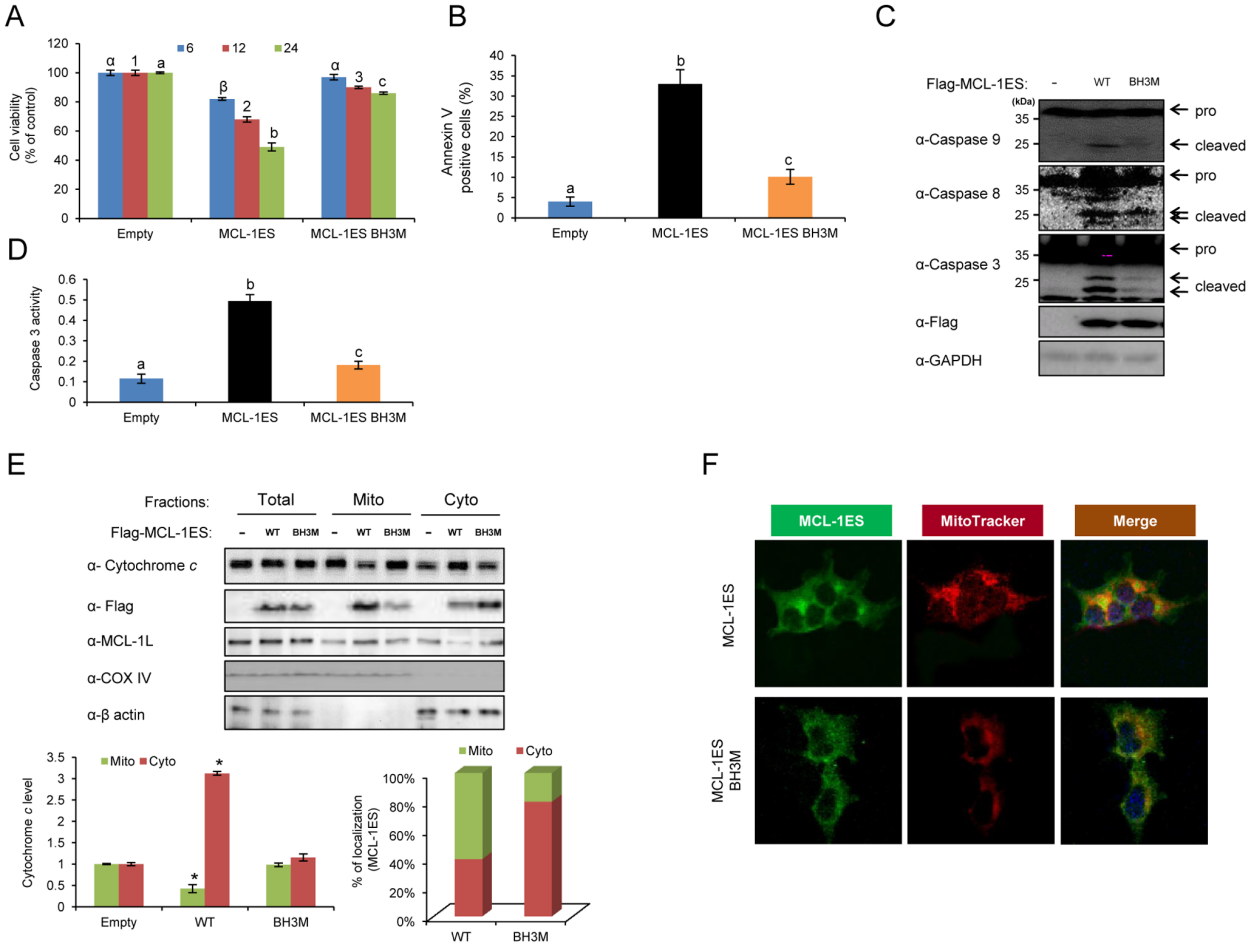
The BH3 domain of MCL-1ES is critical for its apoptotic activity. (A) A time course of cell viability of MCL-1ES and the BH3 mutant (BH3M), (B) Annexin V-positive apoptotic cell analysis, (C) western blot analysis of caspase activation, and (D) caspase 3 activity analysis were performed in 293T cells transfected with WT or the BH3M MCL-1ES mutant as described in the Materials and Methods. The values are expressed as the mean ± SEM of three independent experiments. Different letters denote statistically significant different values (*P*>0.05). (E) Changes in cytochrome *c* release and intracellular localization of BH3M were determined by subcellular fractionation followed by western blot analysis (upper panel). The levels of cytochrome *c* (left graph) and the MCL-1ES protein (right graph) in the mitochondria and the cytoplasm were quantified and are presented (lower panel). (F) Confocal microscopic images of cells overexpressing WT (upper panel) and BH3M MCL-1ES (lower panel) are shown. The cells were stained with an anti-Flag antibody and MitoTracker.

To further define the minimum region responsible for apoptotic activity, seven conserved amino acids (LRRLDIK) in the BH3 domain of MCL-1ES were substituted by alanine (BH3M) (Figure 1A). As expected, this mutation significantly reduced cell death, Annexin V-positive cells, caspase activation, and cytochrome *c* release (Figure 2A–E). Compared to the WT protein, which resides mostly in the mitochondria, the portion of the BH3 mutant protein in the mitochondria decreased, as determined by both subcellular fractionation and confocal microscopy (Figure 2E and F). These results indicate that the BH3 domain of MCL-1ES is important for its mitochondrial localization and apoptosis-inducing activity.

### BAX- and BAK-independent Apoptosis by MCL-1ES

**Figure 3:**
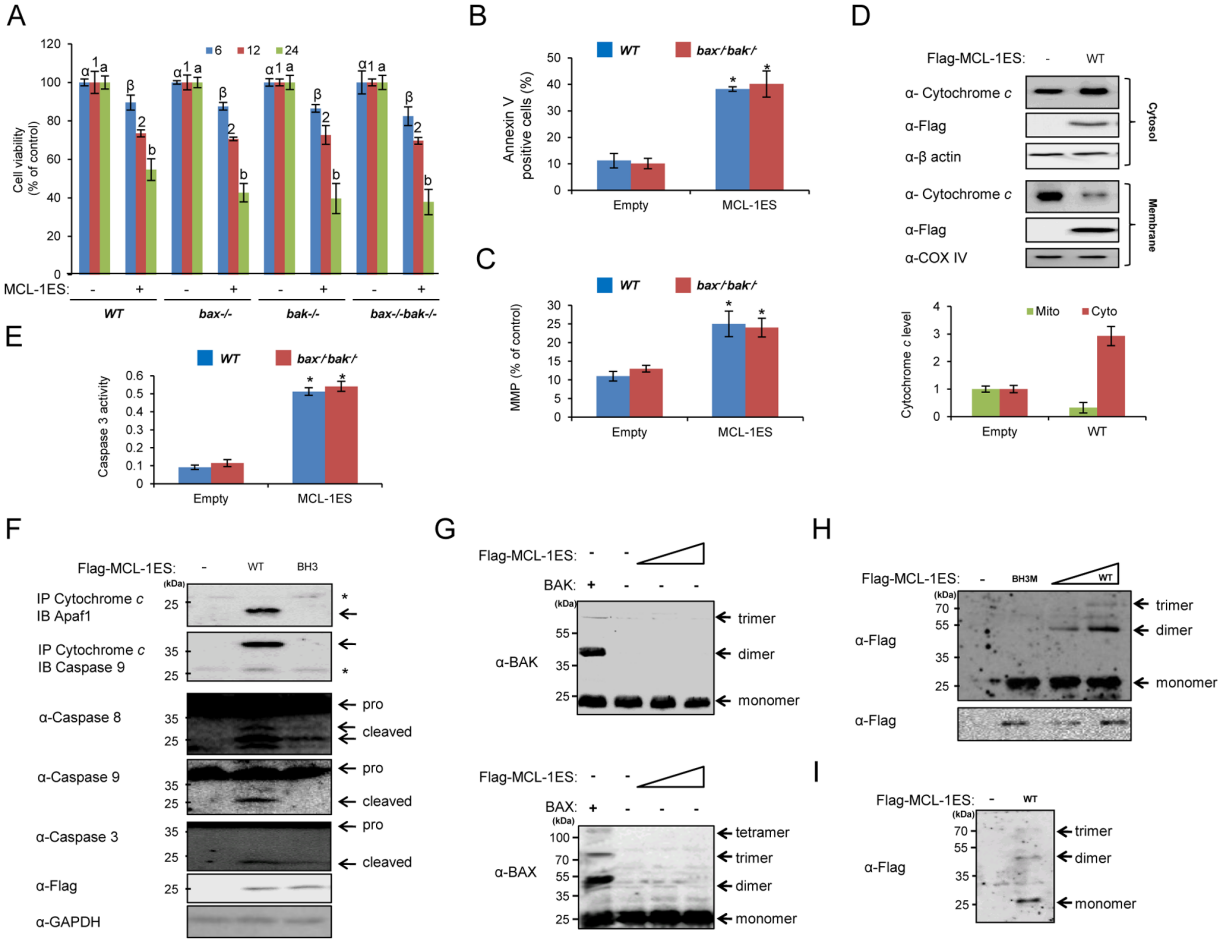
BAK- and BAX-independent apoptosis induced by MCL-1ES via its mitochondrial oligomerization. (A) A time course of cell viability was performed in wild-type (WT), *bak^−/−^*, *bax^−/−^*, and *bax^−/−^bak^−/−^* knockout MEF cells after transfection with WT or BH3 mutant (BH3M) MCL-1ES. (B) A flow cytometry analysis of Annexin V-positive apoptotic cells was performed, and (C) MMP, (D) cytochrome *c* release to cytosol, (E) caspase-3 activity, and (F) formation of apoptosome complexes were determined in *bax^−/−^bak^−/−^* MEF cells transfected with WT or BH3M MCL-1ES as described in the Materials and Methods. The values (mean ± SEM) were determined from three independent experiments. (G) The lack of MCL-1ES-induced BAK or BAX oligomerization was verified in 293T cells. (H) Oligomer formation of MCL-1ES in 293T cells and (I) in *bax^−/−^bak^−/−^* MEF cells was assessed. The heavy membrane fraction was obtained and cross-linked with glutaraldehyde. Oligomer formation was determined by western blot analysis using the appropriate antibodies.

Because BAX and BAK proteins are death effector proteins required for proapoptotic BCL-2 family proteins to elicit mitochondrial cell death, we assessed which effector protein mediates the apoptotic effects of MCL-1ES. Overexpressing MCL-1ES in WT MEF cells resulted in cell death to a similar extent as that observed in 293T cells (Figure 3A). However, MCL-1ES still induced death in cells that were deficient of *bax*, *bak*, or both *bax* and *bak* (Figure 3A), indicating that MCL-1ES induced apoptosis independently of BAX and BAK. Accordingly, MCL-1ES retained its ability to increase Annexin V-positive cells, MOMP, cytochrome *c* release, and caspase 3 activation in *bax* ^−/−^ *bak* ^−/^cells (Figure 3B–E). Moreover, MCL-1ES induced apoptosome complex formation, as demonstrated by the association of apoptotic peptidase activating factor 1 (APAF1), cytochrome *c*, and caspase 9, whereas the BH3 mutant protein failed to stimulate apoptosome formation in *bax* ^−/−^ *bak* ^−/^cells (Figure 3F). In addition, we confirmed that neither BAX nor BAK oligomerized upon MCL-1ES overexpression in 293T cells, whereas ectopically expressing BAX or BAK resulted in oligomerization (Figure 3G). Thus, we hypothesized that MCL-1ES oligomerizes in the mitochondria, leading to MOMP and cytochrome *c* release. Increasing MCL-1ES expression resulted in concentration-dependent oligomerization as its dimer and trimer forms were detected at their respective sizes, 50 and 75 kDa, respectively, as determined by western blot analysis (Figure 3H). However, the BH3 mutant failed to oligomerize (Figure 3H), and truncated MCL-1ES mutants lacking the BH3 domain (containing amino acids 78–197 or 78–173) did not form oligomers (Figure S2). In contrast, MCL-1ES mutants lacking the TM domain but possessing the BH3 sequence (containing amino acids 1–173 and 1–76) retained their abilities to oligomerize (Figure S2). These data suggest that the BH3 domain plays a critical role in the oligomerization of MCL-1ES. In addition, MCL-1ES formed oligomers in *bax* ^−/−^ *bak* ^−/^MEF cells (Figure 3I). Taken together, these results imply that MCL-1ES-induced mitochondrial cell death involves its own oligomerization and not the oligomerization of BAX or BAK.

### MCL-1L as a Critical Mediator of MCL-1ES-induced Apoptosis

**Figure 4:**
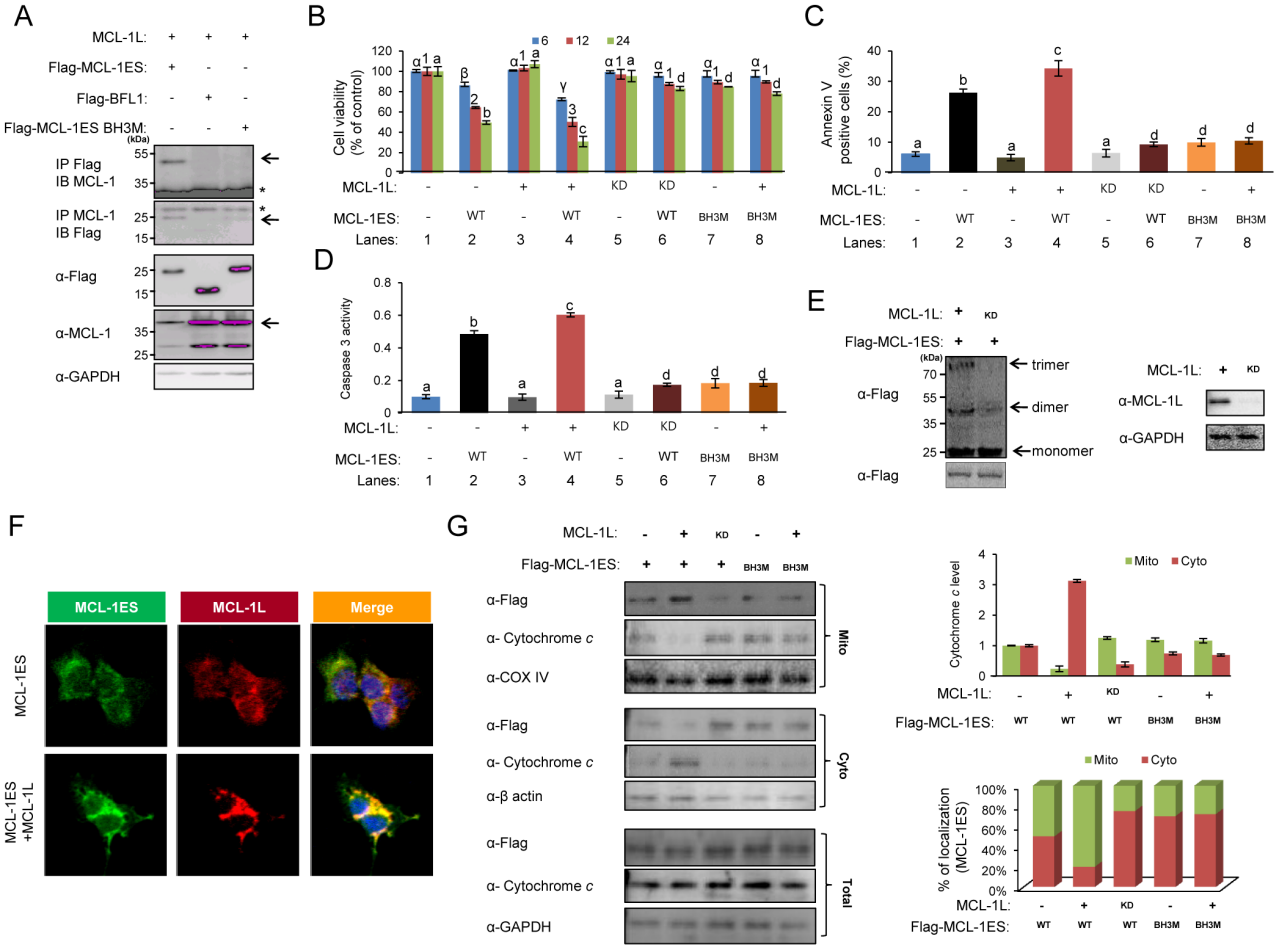
The importance of MCL-1L in MCL-1ES-mediated apoptosis. (A) The interactions between MCL-1L and WT or BH3M MCL-1ES were analyzed by immunoprecipitation in 293T cells followed by western blot analysis. Equal amounts of total protein from cell lysates were used in each lane. (B) A time course of cell viability, (C) Annexin V-positive apoptotic cells, and (D) caspase 3 activity were analyzed in 293T cells that over- or under-expressed MCL-1L after transfecting with WT or BH3M MCL-1ES. Equal amounts of plasmid DNA were used. Data are from two independent experiments performed in triplicate. The percentage of viable 293T cells is expressed as the mean ± SEM. Different letters denote statistically significant different values (*P*>0.05). (E) Changes in MCL-1ES oligomerization were assessed in 293T cells that over- or under-expressed MCL-1L. (F) The influence of MCL-1L on intracellular MCL-1ES localization was determined by immunofluorescent confocal microscopy. 293T cells were stained with anti-MCL-1L and anti-Flag (MCL-1ES) antibodies and visualized with goat anti-mouse IgG or goat anti-rabbit IgG. (G) The influence of MCL-1L on cytochrome *c* release by MCL-1ES and BH3M and the intracellular localization of MCL-1ES and BH3M were determined by subcellular fractionation and western blot analyses (left panel). The quantified results shown in the graphs were from three independent experiments.

In a previous study, we found that the proapoptotic MCL-1ES protein interacts with the antiapoptotic MCL-1L protein, and unconventionally, co-expressing MCL-1L and MCL-1ES augmented the activity of MCL-1ES [8]. MCL-1ES did not interact with any BCL-2 family proteins tested except for MCL-1L in a yeast two-hybrid system (Figure S3). As the BH3 domain is critical for the apoptotic activity of MCL-1ES (Figure 2), we determined whether the interaction between MCL-1ES and MCL-L requires the BH3 sequence. Immunoprecipitation showed that the association of the two MCL-1 proteins is mediated by the BH3 domain of MCL-1ES (Figure 4A). Co-expressing MCL-1L and MCL-1ES potentiated the apoptotic effect of MCL-1ES (lanes 2 vs. 4), and conversely, knocking down MCL-1L attenuated MCL-1ES activity (lanes 2 vs. 6) (Figure 4B). Similar effects on Annexin V-positive cells and caspase 3 activation were observed upon modulation of the MCL-1L level (Figure 4C and D). In contrast, MCL-1L did not potentiate the activity of the BH3 MCL-1ES mutant (lanes 7 vs. 8) (Figure 4B–D). In addition, forced MCL-1L overexpression increased MCL-1ES oligomerization, whereas MCL-1L knockdown decreased oligomer formation (Figure 4E). Confocal microscopic analysis revealed that the mitochondrial localization of MCL-1ES increased in the presence of overexpressed MCL-1L protein (Figure 4F). Western blot analysis of subcellular fractions also showed that the amount of MCL-1ES in the mitochondrial fraction increased upon MCL-1L overexpression, while MCL-1L knockdown inhibited MCL-1ES translocation to the mitochondria (Figure 4G). In contrast, the localization of the BH3 mutant protein, which did not interact with MCL-1L, was not altered by co-expression with the MCL-1L protein (Figure 4G). Accordingly, ectopic expression of MCL-1L stimulated MCL-1ES-induced cytochrome *c* release, while its knockdown inhibited mitochondrial cytochrome *c* liberation by MCL-1ES (Figure 4G). These stimulatory effects of MCL-1L on both MCL-1ES-induced cytochrome *c* release and mitochondrial targeting of MCL-1ES were also confirmed in a cell-free system using recombinant MCL-1 proteins and isolated mitochondria (Figure S4A–C). These results imply that MCL-1L is a critical mediator of MCL-1ES-induced apoptosis and is involved in the mitochondrial localization of MCL-1ES.

## Discussion

Disruption of mitochondrial membrane integrity is the central apoptotic event governed by BCL-2 family proteins. Two BCL-2 family death effectors, BAX and BAK, form oligomers and permeabilize the outer mitochondrial membrane [14]. Most proapoptotic members of the BCL-2 protein family induce mitochondrial cell death using BAX, BAK, or BAX and BAK to induce MOMP [15]. In contrast, we demonstrated that MCL-1ES, a proapoptotic BCL-2 family member, induced apoptosis independently of both BAX and BAK (Figure 3A–E). Furthermore, we determined that MCL-1ES can induce MOMP by its own oligomerization (Figure 3H and I). The distinctive ability of MCL-1ES to form oligomers at the mitochondrial membrane seems to make BAX and BAK dispensable. The secondary structure of MCL-1ES is similar to those of BAX and BAK in that it possesses multiple BH1, BH2, and TM domains, all of which are absent in BH3-only proapoptotic BCL-2 proteins.

Under survival signaling, MCL-1L primarily localizes to the outer mitochondrial membrane and promotes cell survival by sequestering proapoptotic BCL-2 proteins via protein-protein interactions, which prevent BAX- or BAK-induced MOMP [16], [17]. Unlike other BCL-2 proteins that have multiple binding partners, MCL-1ES interacted only with MCL-1L via its BH3 domain (Figure 4A and Figure S3), and other antiapoptotic BCL-2 family proteins, including BFL1, BCL-2, and BCL-xL, did not block MCL-1ES-mediated cell death or affect the interaction between MCL-1ES and MCL-1L (Figure S5A and B). In addition, MCL-1ES effectively induced cell death in MEF cells lacking BH3-only proteins, including *bim* ^−/−^, *noxa* ^−/−^ and *puma* ^−/−^ cells (Figure S5B). Furthermore, MCL-1L promoted the mitochondrial localization of MCL-1ES, whereas MCL-1L overexpression did not alter the localization of a BH3 MCL-1ES mutant protein that did not bind MCL-1L (Figure 4). This result suggests that MCL-1L is important in targeting MCL-1ES to mitochondria. These results may explain why overexpressing a survival protein, MCL-1L, potentiated the apoptotic activity of MCL-1ES, while MCL-1L knockdown inhibited MCL-1ES (Figure 4B–D and G). This distinct relationship between MCL-1L and MCL-1ES is in sharp contrast to the relationship between MCL-1L and MCL-1S; MCL-1L efficiently blocked MCL-1S-induced apoptosis [7]. Unlike MCL-1ES, MCL-1S has a BH3 domain but no BH1, BH2, or TM domains [7], [8]. Thus, the opposite effects of MCL-1L on MCL-1ES and MCL-1S may be a consequence of the BH1, BH2, and TM domains present in MCL-1ES, which may allow it to form oligomers. The precise characteristics of MCL-1ES will become clearer when its crystal structure is analyzed.

The *MCL-1* gene is highly expressed in a variety of cancers [18]. MCL-1 overexpression has been reported in chronic and acute myeloid leukemias, multiple myelomas, hepatocarcinomas, non-small-cell lung cancers, and sarcomas [19]–[21]. MCL-1 is also essential for the survival of leukemic cancers and is closely related to the development of chemoresistance and recurrence in multiple cancers [22]–[25]. Therefore, MCL-1L is an attractive target in treating malignancy, but developing selective inhibitors has been challenging. A recent study by Stewart *et a*l [26] reported that MCL-1 BH3 peptides were potent and exclusive MCL-1 inhibitors [27], [28], which agrees with our observation that the BH3 domain of MCL-1ES binds MCL-1L to inhibit its function. The finding that co-expressing MCL-1ES and MCL-1L not only neutralized MCL-1L activity but also enhanced MCL-1ES apoptotic activity makes MCL-1ES a selective and effective MCL-1L inhibitor in diseases that involve aberrant MCL-1L expression.

## Conclusions

MCL-1ES is distinct from other proapoptotic BCL-2 members in that MCL-1ES induces mitochondrial apoptosis independently of both BAX and BAK. Instead, MCL-1ES formed mitochondrial oligomers, which was followed by MOMP and cytochrome *c* release in the presence of MCL-1L. Therefore, we believe that the significance of this study is two-fold: we increased the understanding of the molecular mechanism by which MCL-1ES induces apoptosis, and we identified MCL-1ES as a selective inhibitor of MCL-1L.

## Supporting Information

Figure S1 **MCL-1ES induces caspase-mediated cell death.** Cell viability was assessed as shown in Figure 1B that the cells were transfected with MCL-1ES and incubated with different concentrations of z-VAD-fmk for 24 h. The values are expressed as mean ± SEM of three determinations.
(TIF)

Figure S2 **The BH3 domain-containing** ***N*** **-terminus region is required for MCL-1ES oligomerization.** Oligomerization was determined as shown in Figure 3G.
(TIF)

Figure S3 **Determination of MCL-1ES interaction with BCL-2 family proteins using a yeast two-hybrid system.** Yeast cells were grown in media containing 15 mM 3-amino-1,2,4-triazole but lacking Trp, Leu, His, and Ade (4D). (−) and (+) denote negative or positive yeast growth and (+++) indicates a strong interaction. MCL-1L and rMCL-1L are from human or rat, respectively.
(TIF)

Figure S4 **MCL-1ES-mediated mitochondrial cytochrome** ***c*** **release in a cell-free system.** Coomassie-stained gels of (A) MCL-1L and (B) MCL-1ES proteins are shown. (C) Isolated mitochondria were incubated with recombinant MCL-1ES and/or MCl-1L proteins. Western blot analysis of cytochrome *c* in mitochondria and supernatant was shown.
(TIF)

Figure S5 **Other BCL-2 family proteins do not play significant roles in MCL-1ES-induced cell death.** (A) The interaction between MCL-1L and MCL-1ES in the absence or presence of BFL1, BCL-2, or BCL-xL was determined by immunoprecipitation in 293T cells followed by western blot analysis. Equal amounts of total protein from cell lysates were used in each lane. (B) The lack of modulation of MCL-1ES-induced cell death by overexpression of BFL1, BCL-2, or BCL-xL was determined in *bax^−/−^bak^−/−^* MEF cells. (C) Cell viability of MCL-1ES with or without MCL-1L overexpression was assessed in *bim* ^−/−^, *noxa* ^−/−^ and *puma* ^−/−^ MEF cells.
(TIF)
