# Supplementary figures and images for: MCL-1ES Induces MCL-1L-Dependent BAX- and BAK-Independent Mitochondrial Apoptosis
Source: PLoS One. 2013 Nov 18;8(11):e79626. doi: 10.1371/journal.pone.0079626 (PMC3832543; doi:10.1371/journal.pone.0079626)

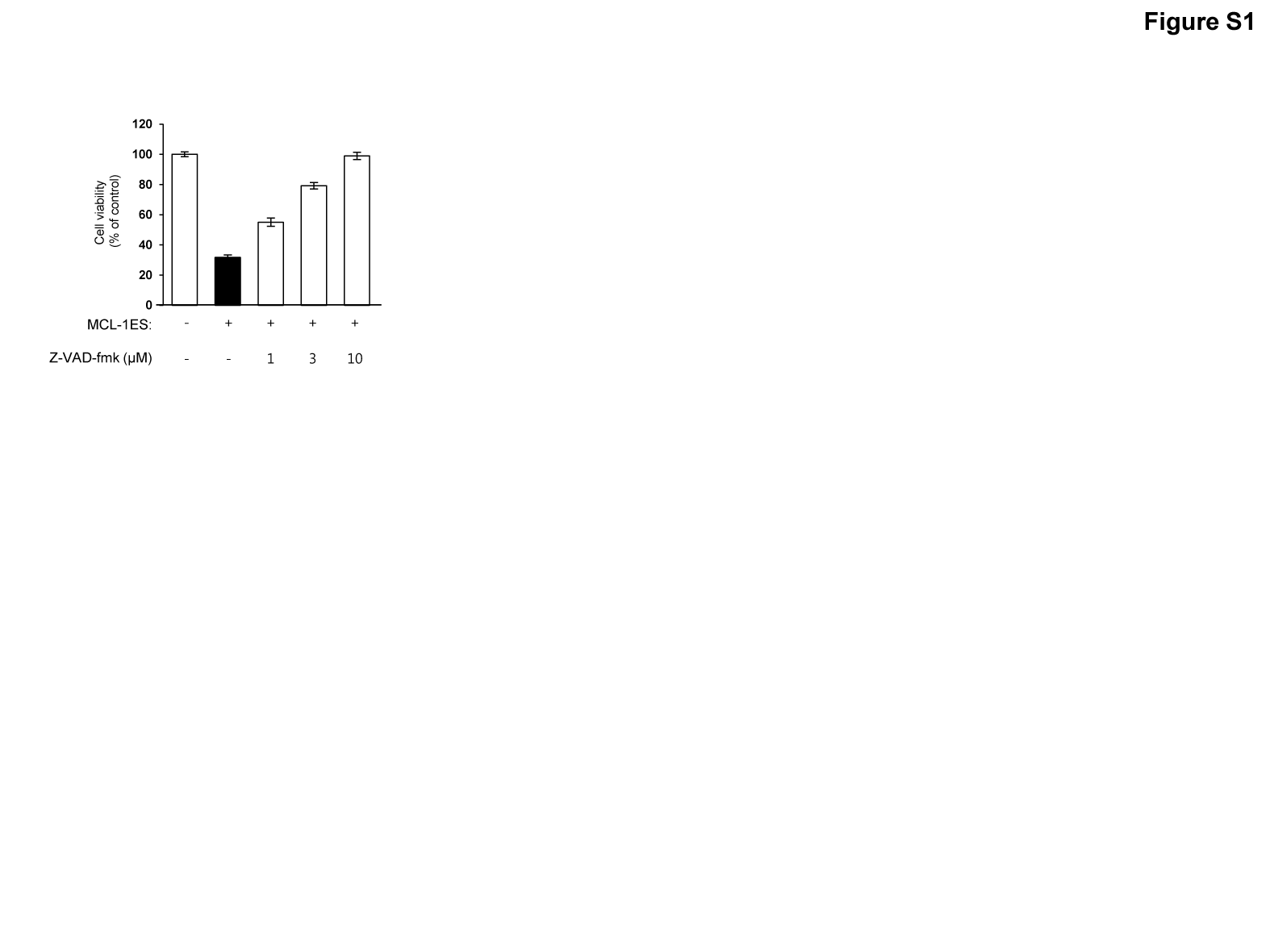

Supplement: Figure S1 — MCL-1ES induces caspase-mediated cell death. Cell viability was assessed as shown in Figure 1B that the cells were transfected with MCL-1ES and incubated with different concentrations of z-VAD-fmk for 24 h. The values are expressed as mean ± SEM of three determinations. (TIF) [file pone.0079626.s001.tif]

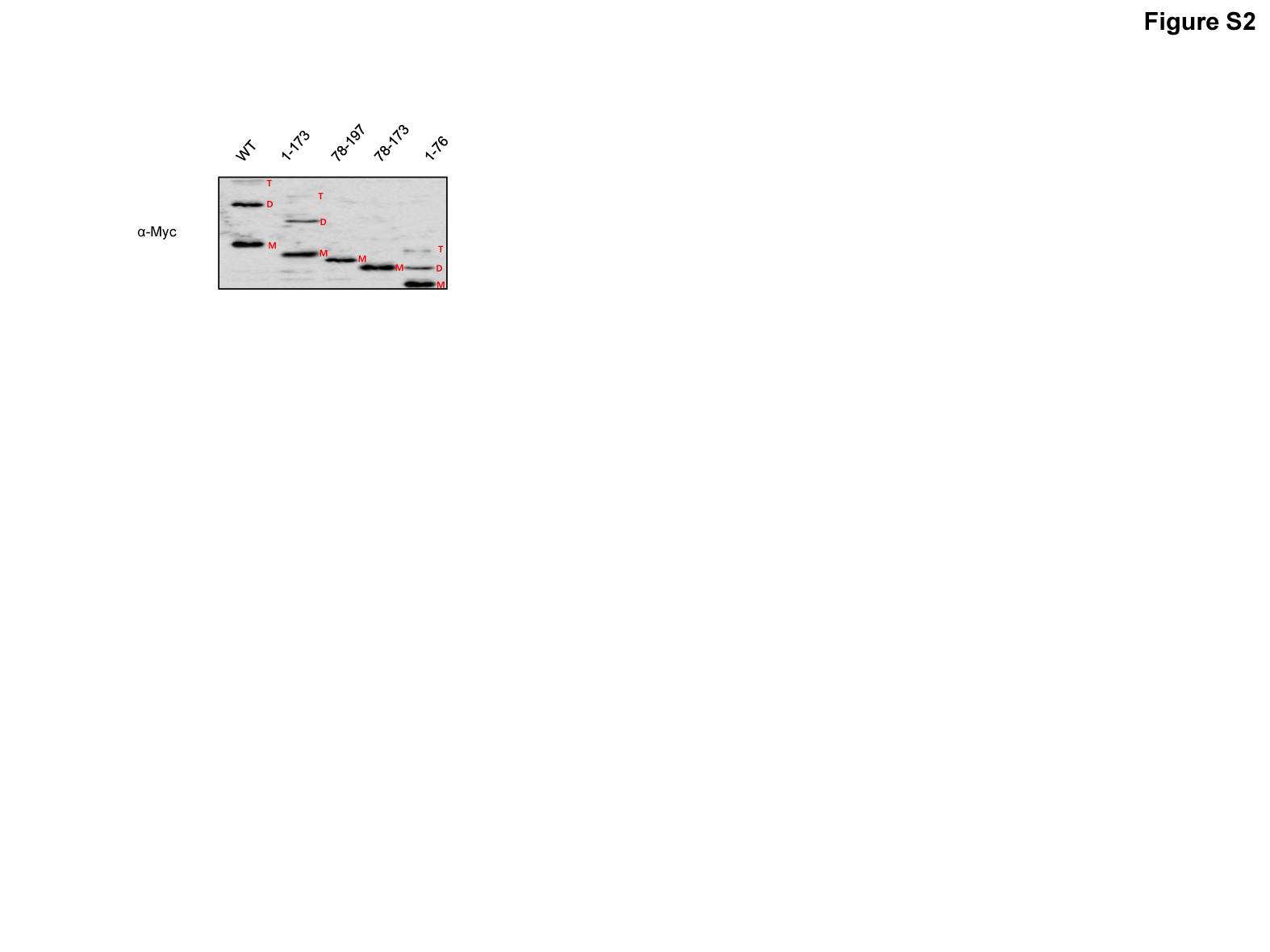

Supplement: Figure S2 — The BH3 domain-containing N -terminus region is required for MCL-1ES oligomerization. Oligomerization was determined as shown in Figure 3G. (TIF) [file pone.0079626.s002.tif]

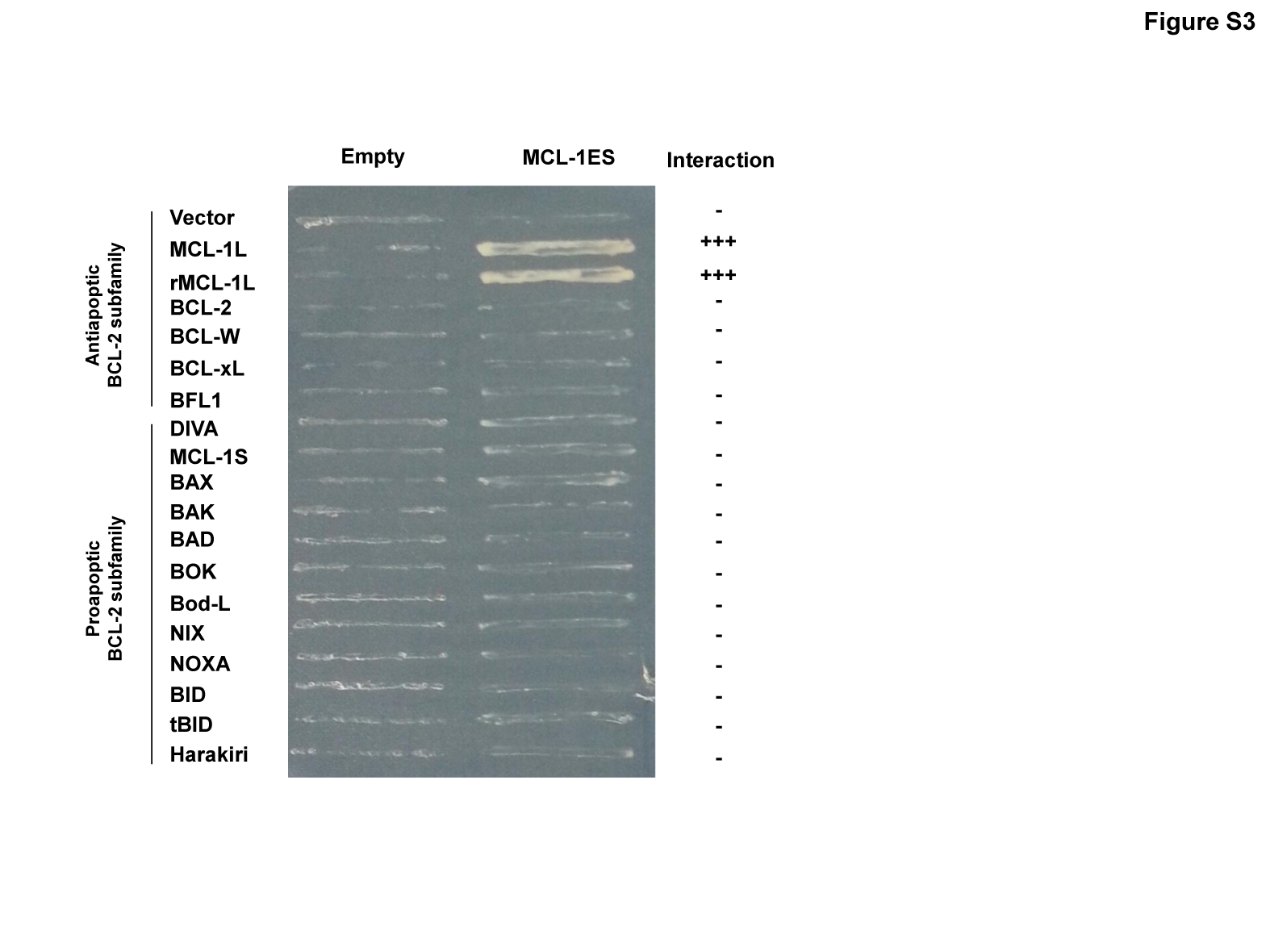

Supplement: Figure S3 — Determination of MCL-1ES interaction with BCL-2 family proteins using a yeast two-hybrid system. Yeast cells were grown in media containing 15 mM 3-amino-1,2,4-triazole but lacking Trp, Leu, His, and Ade (4D). (−) and (+) denote negative or positive yeast growth and (+++) indicates a strong interaction. MCL-1L and rMCL-1L are from human or rat, respectively. (TIF) [file pone.0079626.s003.tif]

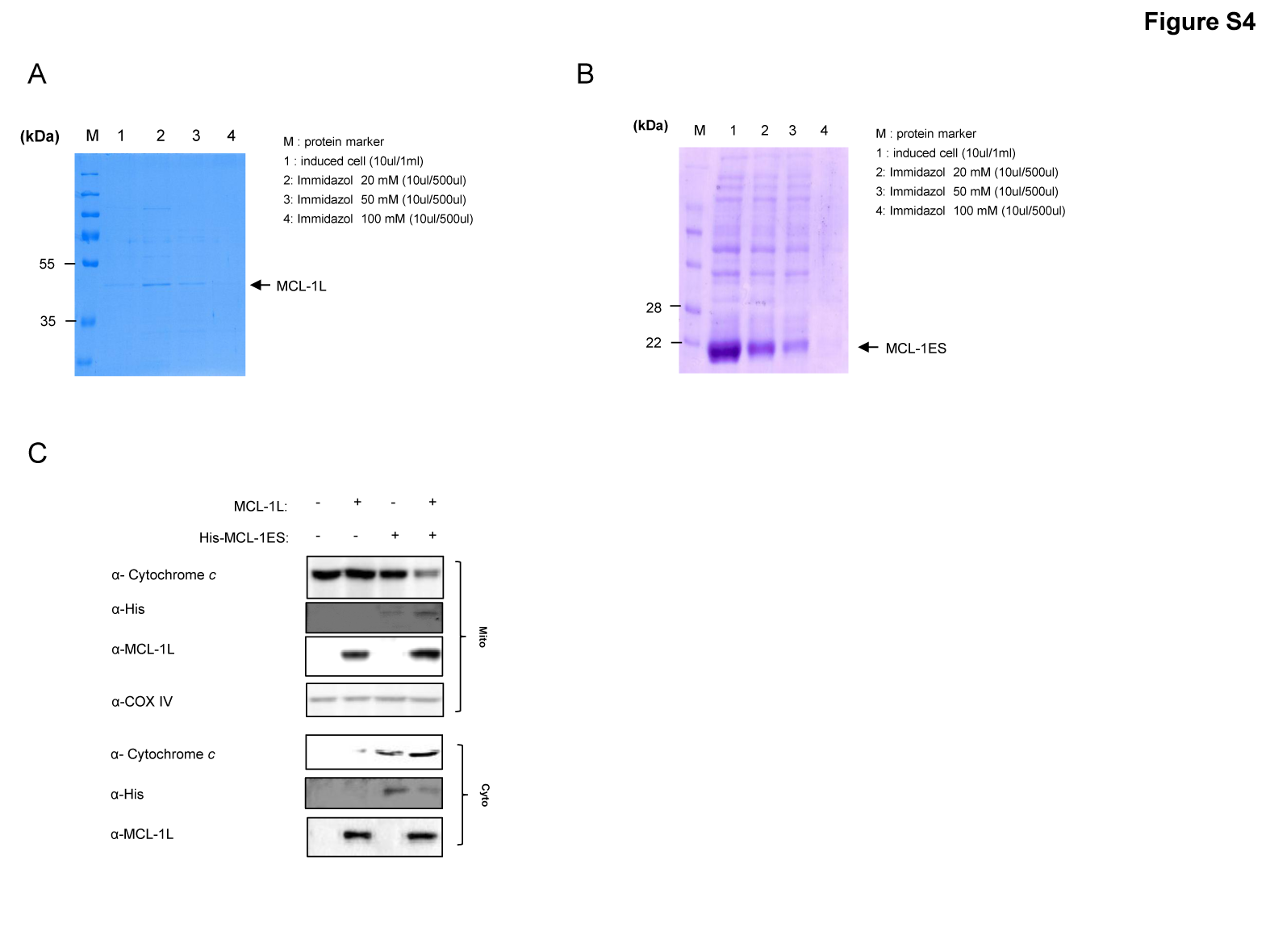

Supplement: Figure S4 — MCL-1ES-mediated mitochondrial cytochrome c release in a cell-free system. Coomassie-stained gels of (A) MCL-1L and (B) MCL-1ES proteins are shown. (C) Isolated mitochondria were incubated with recombinant MCL-1ES and/or MCl-1L proteins. Western blot analysis of cytochrome c in mitochondria and supernatant was shown. (TIF) [file pone.0079626.s004.tif]

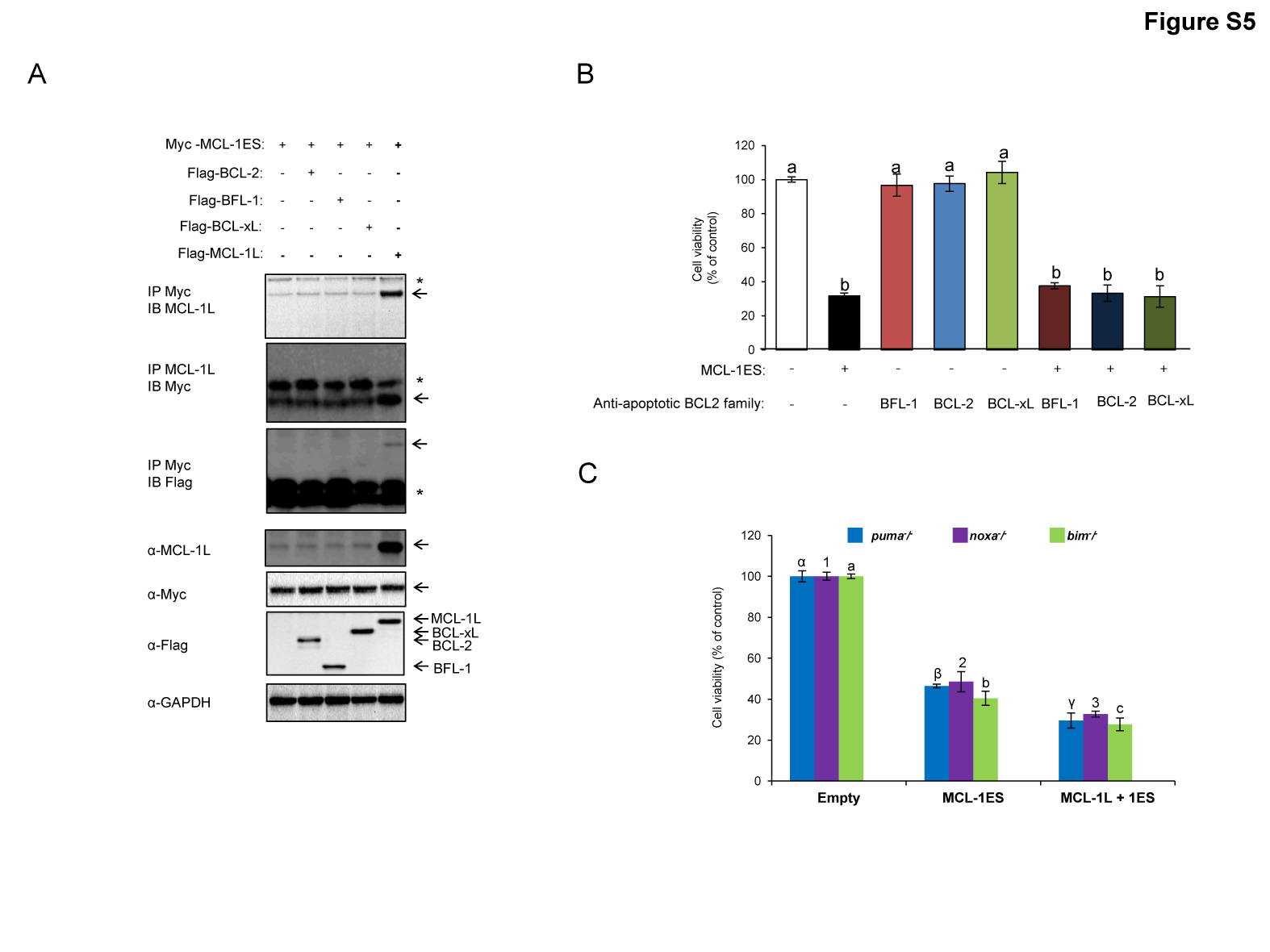

Supplement: Figure S5 — Other BCL-2 family proteins do not play significant roles in MCL-1ES-induced cell death. (A) The interaction between MCL-1L and MCL-1ES in the absence or presence of BFL1, BCL-2, or BCL-xL was determined by immunoprecipitation in 293T cells followed by western blot analysis. Equal amounts of total protein from cell lysates were used in each lane. (B) The lack of modulation of MCL-1ES-induced cell death by overexpression of BFL1, BCL-2, or BCL-xL was determined in bax−/−bak−/− MEF cells. (C) Cell viability of MCL-1ES with or without MCL-1L overexpression was assessed in bim −/−, noxa −/− and puma −/− MEF cells. (TIF) [file pone.0079626.s005.tif]
